# Voltammetric Determination of Levodopa Using Mesoporous Carbon—Modified Screen-Printed Carbon Sensors

**DOI:** 10.3390/s21186301

**Published:** 2021-09-20

**Authors:** Dorin Dăscălescu, Constantin Apetrei

**Affiliations:** Department of Chemistry, Physics and Environment, Faculty of Sciences and Environment, “Dunărea de Jos” University of Galaţi, 47 Domnească Street, 800008 Galaţi, Romania; dorin.dascalescu@ugal.ro

**Keywords:** levodopa, screen-printed sensor, cyclic voltammetry, ordered mesoporous carbon, dietary supplements

## Abstract

Levodopa is a precursor of dopamine, having important beneficial effects in the treatment of Parkinson’s disease. In this study, levodopa was accurately detected by means of cyclic voltammetry using carbon-based (C-SPCE), mesoporous carbon (MC-SPCE) and ordered mesoporous carbon (OMC-SPCE)-modified screen-printed sensors. Screen-printed carbon sensors were initially used for the electrochemical detection of levodopa in a 10^−3^ M solution at pH 7.0. The mesoporous carbon with an organized structure led to better electroanalysis results and to lower detection and quantification limits of the OMC-SPCE sensor as compared to the other two studied sensors. The range of linearity obtained and the low values of the detection (0.290 µM) and quantification (0.966 µM) limit demonstrate the high sensitivity and accuracy of the method for the determination of levodopa in real samples. Therefore, levodopa was detected by means of OMC-SPCE in three dietary supplements produced by different manufacturers and having various concentrations of the active compound, levodopa. The results obtained by cyclic voltammetry were compared with those obtained by using the FTIR method and no significant differences were observed. OMC-SPCE proved to be stable, and the electrochemical responses did not vary by more than 3% in repeated immersions in a solution with the same concentration of levodopa. In addition, the interfering compounds did not significantly influence the peaks related to the presence of levodopa in the solution to be analyzed.

## 1. Introduction

The most important component of *Mucuna pruriens* seeds is levodopa (L-DOPA, L-3,4-dihydroxyphenylalanine), which is currently the standard medication for the treatment of Parkinson’s disease [1]. This disease is a progressive neurological disorder that is triggered when the brain fails to produce enough dopamine [2,3]. Dopamine deficiency induces a number of neuromuscular dysfunctions, such as tremour, muscle stiffness, slowing of movements (bradykinesia) and loss of balance [4,5].

The progressive degeneration of dopaminergic neurons in the mesolimbic and mesocortical pathways, along with the presence of Lewy corpuscles in the mesencephalon, are the main causes of the first symptoms related to Parkinson’s syndrome [6,7].

There exists a parallel alteration of cognitive functions, due to the prefrontal cortex and hippocampus being affected [8,9]. Since dopamine cannot be administered directly because it does not penetrate the blood–brain barrier, L-DOPA is the appropriate precursor for reducing the symptoms of Parkinson’s disease [10]. Taking supplements based on this amino acid could prevent neurodegenerative diseases, its beneficial effects extending to stress and libido problems. The use of novel pharmaceutical technologies for the controlled release of active compounds can increase the efficiency of such products in clinical practice [11].

Considering that food supplements are commercial products, which may be taken without medical recommendations, this aspect could lead to improper dosing. *Mucuna pruriens* extract contains L-DOPA and it is found in several food supplements. Given the biological effects of L-DOPA, its determination by various methods has attracted the interest of numerous research teams. Over time, various techniques for the detection of levodopa have been used, such as high-performance liquid chromatography [12], spectrophotometry [13], chemiluminescence [14] and electrochemical methods [15,16].

Due to the fast response, the simple equipment used and the possibility of miniaturizing detection instruments without the pre-treatment of samples [17,18], electrochemical methods do not have certain limitations specific to classical methods. Moreover, electrochemical methods are very useful for the evaluation of pharmaceuticals and biological samples, due to their high portability and sensitivity. Modifications of screen-printed electrodes [19], carbon paste [16] or glassy carbon [20,21] by using nanomaterials such as graphene and graphene oxide, carbon nanotubes, metal nanoparticles, nanocomposites and conductive polymers may allow the evaluation of numerous types of samples, at the nanomolar level [22].

Carbon nanomaterials are often used and are primarily synthesized by three main techniques: arc discharge, chemical vapor deposition (CVD) and laser ablation/vaporization, each of the methods having advantages and limitations [23].

Regarding the mesoporous carbon materials, their synthesis was initially produced using spherical solid gel as the template, in several stages, including: preparation of silica gel with controlled pore structure, impregnation/infiltration of the silica template with monomer or polymer precursors, cross-linking and carbonization of the organic precursors and dissolution of the silica template [24,25]. Methods for the synthesis of ordered mesoporous carbon also have certain limitations, if they would involve a high temperature. High-temperature methods using various carbon sources were found to lead to the collapse of the pore structure. This problem can be avoided by using aromatic precursors, such as benzene [26]. Subsequently, Kyotani et al. developed a successful method for the synthesis of mesoporous carbon, using zeolites as template materials, following two steps: impregnation followed by chemical vapor deposition [27].

For the development of organized mesoporous carbon materials, ionic surfactants, block copolymers, and neutral amines were used as structure-directing agents [24]. For example, chemical vapor deposition (CVD) is a method often applied to the synthesis of ordered mesoporous carbon and some researchers have used the MCM-48 aluminosilicate as a template and acetylene as a carbon precursor [28].

Mesoporous carbon materials have attracted the attention of researchers in recent years due to their uniform pore structure, high specific surface area, good conductivity, regular pore sizes and chemical inertia [29]. In particular, ordered mesoporous carbon has multiple active sites that facilitate the transfer of electrons/protons, allowing efficient mass transport [30]. Additionally, the high thermal stability, flexible framework composition made it suitable for investigating the electrochemical behavior of several substances [31]. Over time, sensors based on mesoporous carbon or ordered mesoporous carbon have shown excellent electrocatalytic capacity, low detection limits, good sensitivity and wide linearity intervals in the detection of several analytes such as L-cysteine [31], morphine [29], tryptophan [30], hydrazine [32] or 4-nitrophenol [33].

Mesoporous carbon materials are of great interest in the field of sensors and therefore, these materials were used in this study in order to increase the sensor performance characteristics. The main novelty of this research work was the electrochemical and morphological characterization of the screen-printed sensors modified with mesoporous carbon and ordered mesoporous carbon, respectively, and the development of an electroanalytical method for sensitive levodopa determination.

The present study aims to characterize and analyze the electroanalytical performance of three screen-printed carbon sensors modified with mesoporous carbonaceous materials (mesoporous carbon and ordered mesoporous carbon) and detecting L-DOPA in food supplements by means of cyclic voltammetry. The results obtained by using the sensor with the best analytical performance will be validated using the FTIR spectrometric method.

## 2. Materials and Methods

### 2.1. Reagents and Solutions

Monosodium phosphate, disodium phosphate and potassium ferrocyanide were purchased from Sigma-Aldrich (St. Louis, MO, USA). Phosphate buffer solution (PBS) was obtained by dissolving NaH_2_PO_4_ and Na_2_HPO_4_ in ultrapure water produced by using a Milli-Q system Millipore, Bedford, MA, USA. The pH of the solutions was checked and corrected by using the Inolab pH meter 7310 (WTW, Weilheim, Germany).

10^−1^ M phosphate buffer (PBS) pH 7.0 and 10^−3^ M-PBS 10^−1^ M potassium ferrocyanide solution were used to characterize the sensors.

L-DOPA of analytical purity was purchased from Sigma-Aldrich. By dissolving the proper amount of L-DOPA in 10^−1^ M PBS (pH = 7.0) a stock solution with a concentration of 10^−3^ M was obtained.

In order to prepare the solutions from real samples, the following steps were followed: the contents of several capsules were released from the shell, 0.015 g, 0.035 g and 0.055 g of the powder mixture, were weighed, these quantities being dispersed in 50 mL of phosphate buffer 10^−1^ M, pH 7.0. The resulting solution was ultrasonicated using an ultrasonic bath (Elmasonic Carl Roth GmbH, Karlsruhe, Germany) for dissolving and homogenization. The samples were analyzed with the sensors after filtering through filter paper (clear solutions).

Potassium bromide of analytical purity (Fluka) was used for preparing the samples to be analyzed by using the FTIR spectrometric method.

In the case of interference studies, several amino acids purchased from Sigma-Aldrich were used: L-tyrosine, L-tryptophan, and L-phenylalanine.

### 2.2. Electrodes and Equpiment

An EG&G potentiostat/galvanostat (Princeton Applied Research, Oak Ridge, TN, USA), model 263 A with an electrochemical cell (Princeton Applied Research, Oak Ridge, TN, USA) was used to characterize the sensors and to optimize their electrochemical signal. The recording and processing of experimental data were performed by using the ECHEM software. The electrochemical cell was composed of three electrodes, namely the reference electrode (Ag/AgCl), the working electrode and the counter electrode (a Pt wire). The working electrodes were: 1. a screen-printed carbon electrode (C-SPCE), 2. a mesoporous carbon modified screen-printed carbon electrode (MC-SPCE) and 3. an ordered mesoporous carbon modified screen-printed carbon electrode (OMC-SPCE). 

The screen-printed sensors used were purchased from Metrohm DropSens (Oviedo, Spain), https://www.dropsens.com/ (accessed on 31 August 2021). The screen printing technique is a method often used, which allows the manufacture of microchips in series, with different thicknesses, at very low costs [34].

The working electrodes, unmodified and modified with carbonaceous materials, have 4 mm in diameter screen prints on a ceramic substrate with dimensions L33 × W10 × H0.5 mm [35]. Although the formulation of printing inks is considered by the manufacturer as proprietary information, it is known that these inks are prepared from carbon precursor (C, MC, OMC in this case), as electrodic material, a polymeric binder increases the affinity of the ink for the substrate in terms of adhesion properties and mechanical strength and a solvent to improve the viscosity of the ink [36].

The morphological characterization of sensors was carried out by a scanning electron microscope (SEM) (FlexSEM 1000, Hitachi, Japan).

The FTIR analysis was performed with the Bruker ALPHA FT-IR spectrometer (BrukerOptik GmbH, Ettlingen, Germany) using the OPUS software (BrukerOptik GmbH, Ettlingen, Germany).

### 2.3. The Real Samples Analyzed

The products chosen for analysis were purchased from health food stores, after checking their composition. The first product is Mucuna Pruriens Bio Powder 125 g (Bio Raw Foods, New Delhi, India). It contains powder obtained by grinding *Mucuna pruriens* seeds. The consumption of these seeds helps in regaining a good state of being, in treating Parkinson’s disease or the lack of libido. The second product is L-Dopa Mucuna Pruriens (Haya Labs, Washington, CA, USA), with natural Mucuna seed extract. This product has a beneficial effect on mood, relieves depression and supports brain function during physical and mental stress. The capsule also contains rice flour, magnesium stearate and vegetable cellulose. The third food supplement is DOPA Mucuna (Now Foods, New York, NY, USA), which additionally contains cellulose, stearic acid and magnesium stearate.

### 2.4. Methods of Analysis

Cyclic voltammetry was applied in all the analysis stages, therefore it was obtained information about the L-DOPA oxidation-reduction process and the reaction mechanism, its identification in different solutions, the calculation of the diffusion coefficient and preparation of the calibration curve. Electrochemical measurements were performed in the potential range between −0.4 and +1.3 V in the preliminary studies or ranging from −0.4 to +0.7 V in the case of L-DOPA detection, applying scan rates among 0.1 and 1.0 V·s^−1^.

FTIR spectrometric measurements were performed in the range 4000–500 cm^−1^, using the ATR (attenuated total reflectance) sampling mode. In order to obtain optimal results, the ZnSe crystal was cleaned with ultrapure water and isopropanol between measurements. The background used was the air.

## 3. Results and Discussion

### 3.1. Preliminary Stages

**Figure 1:**
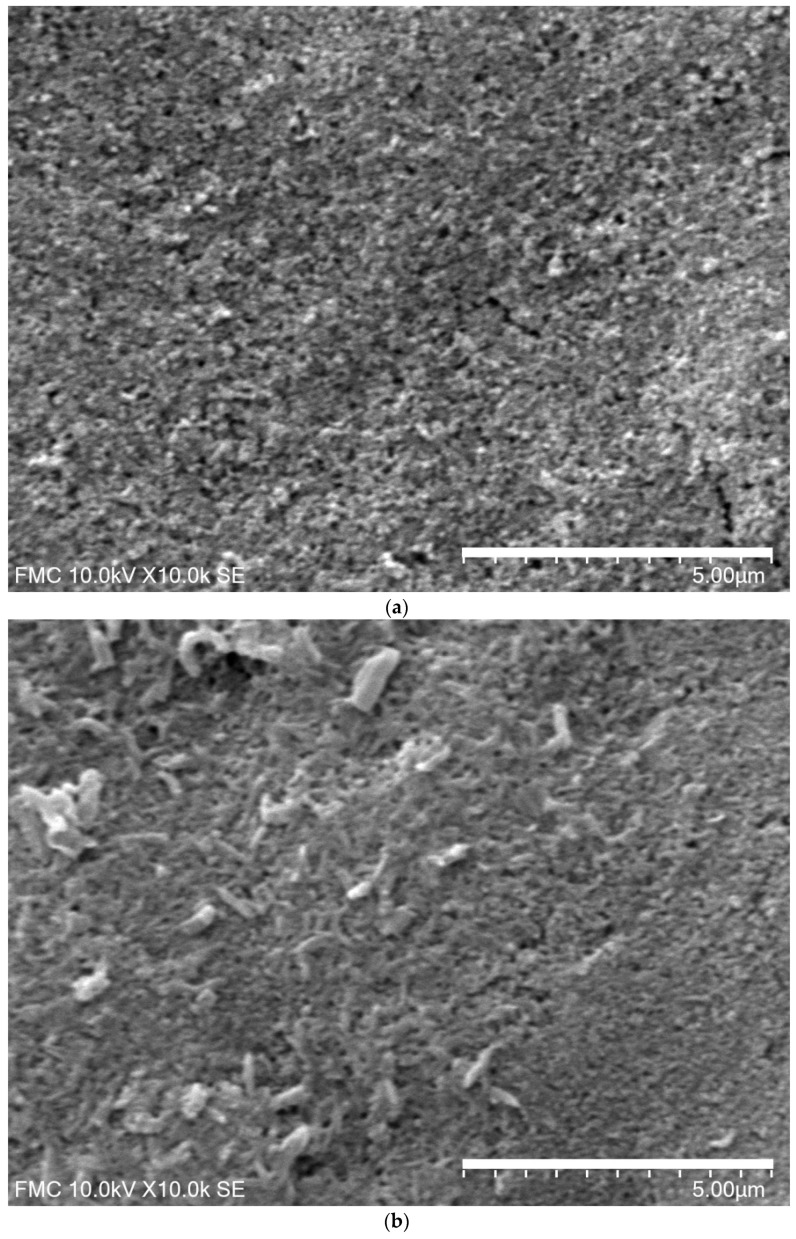
The SEM image of sensors surface (**a**) MC-SPCE and (**b**) OMC-SPCE.

The sensors were initially characterized by scanning electron microscopy in order to assess the differences among carbonaceous materials immobilized on the surface. The surface images of MC-SPCE, OMC-SPCE obtained by SEM were presented in Figure 1.

It can be observed different morphological structures of the sensitive materials deposited on the electrode surface. Comparing the images, the surface of OMC-SPCE is grainier and the superficial interactions useful in the electrochemical processes seem to be more favorable. It will be demonstrated in the electrochemical studies when the active surface will be determined.

**Figure 2:**
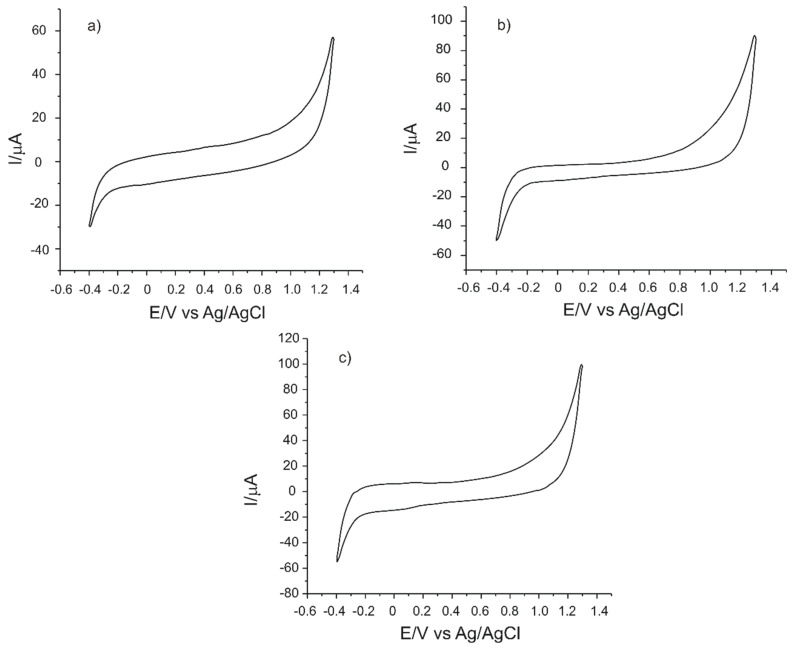
CVs of (**a**) C-SPCE, (**b**) MC-SPCE, (**c**) OMC-SPCE recorded in 0.1 M PBS solution. Scan rate was 0.1 V·s^−1^.

The electrochemical behavior of sensors towards support electrolyte and typical redox probe (potassium ferrocyanide) was studied with the purpose of determining the influence of mesoporous materials in the sensing properties. Then, the three sensors, C-SPCE, MC-SPCE and OMC-SPCE, were characterized by using cyclic voltammetry in a 0.1 M PBS solution, pH 7.0 at a 0.1 V·s^−1^ scan rate. By optimization of the electrochemical parameters, stable signals of the sensors were obtained in the potential range between −0.4 and +1.3 V. Figure 2 shows the cyclic voltammograms of the three sensors immersed in a 0.1 M PBS solution at a scan rate of 0.1 V·s^−1^.

The cyclic voltammograms recorded by the three sensors do not show anodic or cathodic peaks, an aspect that confirms the fact that the active surface of these electrodes is not contaminated and that the electrochemical measurements will not be influenced by interferences present on the electrode surface. In the case of OMC-SPCE, the background current is very low, lower than in the case of the other sensors, suggesting some advantages of the material with the organized structure on the sensor surface.

In the next stage, the electrochemical behavior of the three screen-printed electrodes in a 1.0 × 10^−3^ M potassium ferrocyanide solution-0.1 M PBS was analyzed, this being a reference redox system for the evaluation of carbon-based materials [37,38]. In each case, a pair of well-defined peaks of different features was observed. The anodic peak occurs as a result of the ferrocyanide ion oxidation, whereas the cathodic peak is the result of the ferricyanide ion reduction at the working electrode surface.

**Figure 3:**
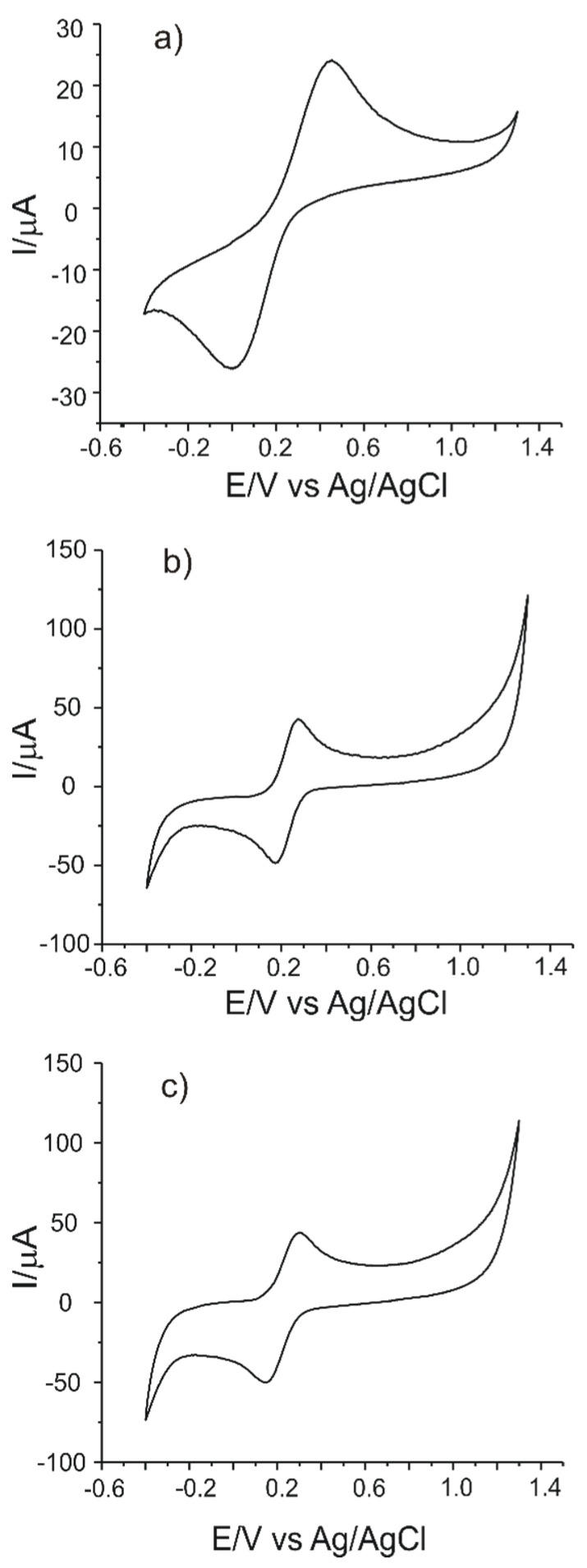
CVs of (**a**) C-SPCE, (**b**) MC-SPCE, (**c**) OMC-SPCE recorded in 1.0 × 10^−3^ M K_4_[Fe (CN)_6_]-0.1 M PBS solution.

Figure 3 shows the cyclic voltammograms of the three sensors immersed in the potassium ferrocyanide solution and PBS 0.1 M at 0.1 V·s^−1^ scan rate.

The electrochemical responses were analyzed in order to prove the influence of mesoporous carbon materials on the sensor responses. Table 1 presents the electrochemical parameters of the screen-printed electrodes obtained from the cyclic voltammograms presented in Figure 1.

Both the intensity of the anodic peak and the intensity of the cathodic peak have higher values in the case of OMC-SPCE, with a small difference as compared to MC-SPCE, but almost double as compared to C-SPCE. This proves that the L-DOPA redox process is favored by the presence of mesoporous carbon, which increases the rate of electron transfer [39].

The half-wave potential (E_1/2_) depends on the nature of the electroactive species and on the composition of the electrolyte solution. In this case, E_1/2_ has similar values for the three electrodes, i.e., around 0.22 V. This result is closer to other values obtained under experimental conditions similarly reported in the literature [37].

OMC-SPCE and MC-SPCE showed a higher degree of reversibility because the separation between the anodic and the cathodic peak was smaller than in the case of C-SPCE. Nevertheless, for a reversible, single-electron transfer reaction, ΔE has a high value [37]. The I_c_/I_a_ ratio was optimal for the three sensors used, with values close to the ideal value 1. This may be due to the uniform pore structure, the larger active surface (demonstrated in the next section) and the numerous active sites.

According to the literature, OMC presents the excellent features of carbonaceous materials in the electroanalysis having high surface area and open-pore structure, which facilitate the increase of the electron-transfer rate [40,41], which could explain the superior electrochemical characteristics of OMC-SPCE.

### 3.2. Determination of the Active Surface of C-SPCE, MC-SPCE, OMC-SPCE

In order to calculate the active surface of the three electrodes, cyclic voltammograms were recorded at different scan rates, in the range 0.1–1 V·s^−1^. The magnitude of the active surface is related to the sensitivity of the sensors and is an indicator of the sensitive properties of the materials immobilized on the receptor element of the sensor.

**Figure 4:**
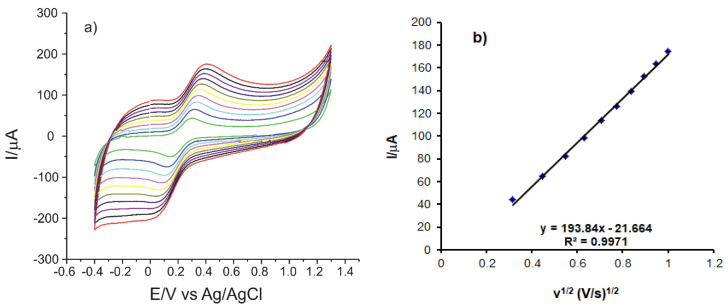
(**a**) CVs recorded by OMC-SPCE in a K_4_[Fe (CN)_6_] 1.0 × 10^−3^ M-PBS 0.1 M solution at different scan rates (0.1–1.0 V·s^−1^) and (**b**) Linear dependence between the anodic current and the square root of scan rate.

When the scan rate increases, there is a slight shift of the potentials to higher potentials (anodic peak) and to lower potentials (cathodic peak) as well as an increase in the potassium ferrocyanide oxidation–reduction peaks (Figure 4a).

By recording the CVs for the three sensors at scan rates varying between 0.1 and 1.0 V·s^−1^ linearity was established between I_pa_ and the square root of the scan rate, the linear equation.

The results, which were similar for the three sensors, are presented in Table 2.

Considering the results obtained, the fact may be stated that the process which takes place at the surface of the electrodes is controlled by the diffusion of the electroactive species. Therefore, the Randles–Sevcik equation was used to calculate the area of the active surface for each sensor [42,43]

I_pa_ = 268,600 × n^3/2^ × A × D^1/2^ × C × v^1/2^ (1)

I_pa_ represents the anodic peak current (A)
n is the number of electrons transferred in the redox process, 1 in this case
A is the electrode area (cm^2^)
D represents the diffusion coefficient (cm^2^ s^−1^)
C is the concentration (mol cm^−3^)
v is the scan rate (V·s^−1^)
The diffusion coefficient of the ferrocyanide ion is D = 7.26 × 10^−6^ cm^2^·s^−1^ [42]

Using the slope of the I_pa_ equation as a v^1/2^ function, the areas of the active surface of the electrodes were calculated. The results are presented in Table 2.

As it may be seen in Table 2, the OMC-SPCE shows the largest active area, which explains the defined appearance of the observed peaks as well as the higher peak intensities. OMC-SPCE has a 2.12365 roughness factor, which could be due to the organized distribution of mesoporous carbon on the surface of the support electrode and on the large pore size [41], which leads to increased electron transfer and, thus, to a higher sensitivity. MC-SPCE also has a roughness factor close to 2, which makes it suitable for the subsequent determination of the analyte to be analyzed. The roughness factor was calculated as the ratio between the area of the active surface and the geometric area of the electrode [42].

### 3.3. Electrochemical Responses of Sensors in L-DOPA Solutions

In order to determine qualitatively and quantitatively L-DOPA, the three carbon-based sensors, C-SPCE, MC-SPCE and OMC-SPCE were used. The oxidation–reduction process of the compound envisaged in a solution of L-DOPA 10^−3^ M was studied for these determinations. The signal was stabilized after recording three cycles in the potential range from −0.4 to +0.7 V.

In the next stage, pH optimization studies were performed. The electrochemical behavior of levodopa was investigated at different pH values in the range 5.5–8.0 with all sensors. Each sensor was immersed, in turn, in solutions 10^−3^ M of levodopa (PBS support electrolyte) with different pHs: 5.5, 6.0, 6.5, 7.0, 7.5 and 8.0. The recorded cyclic voltammograms showed the increase of the anodic peak corresponding to the oxidation of levodopa when the pH increase up to pH 7.0. Starting with pH 7.5, the current of the anodic decrease. At pH 8.0 the current is significantly lower comparing with the value at pH 7.0. These results are consistent with those reported in the literature, maximum peak currents were observed at pH close to neutral value [44,45,46,47].

**Figure 5:**
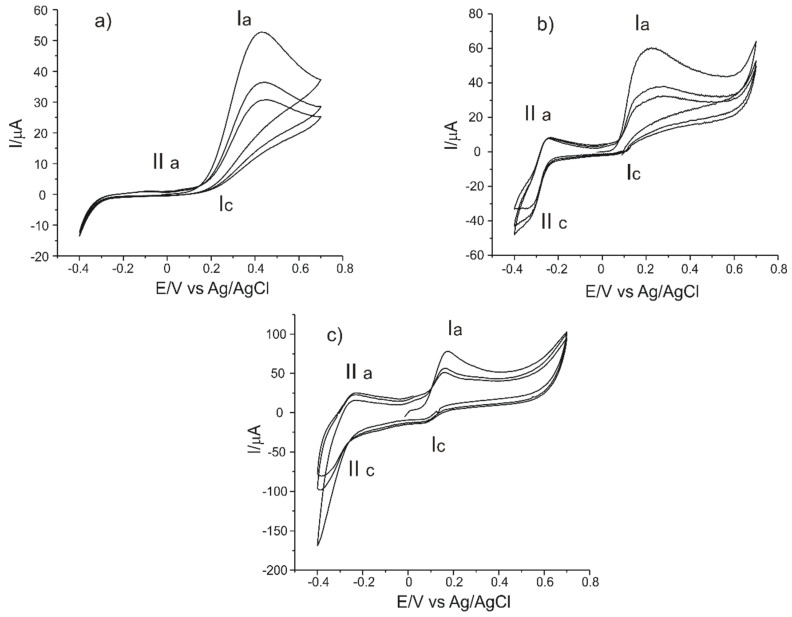
Cyclic voltammograms (three successive cycles) recorded by: (**a**) C-SPCE, (**b**) MC-SPCE and (**c**) OMC-SPCE immersed in 10^−3^ M L-DOPA solution for a 0.1 V·s^−1^ scan rate.

Taking into account these results, pH 7.0 was the optimal value of the background electrolyte for the detection of levodopa with carbonaceous-based sensors used in this study. Figure 5 shows the cyclic voltammograms of the three electrodes in 10^−3^ M-PBS 10^−1^ M solution (pH 7.0) in the process of stabilizing the electrochemical signal. Three cycles were necessary in order to stabilize the sensor responses in 10^−3^ M-PBS 10^−1^ M solution (pH 7.0).

**Figure 6:**
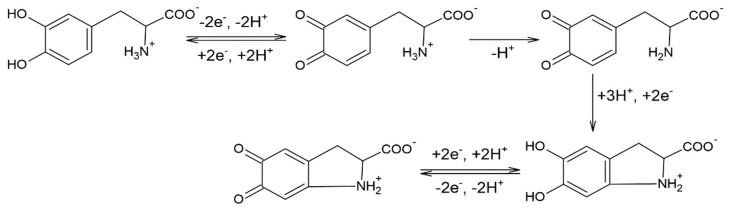
Mechanism of L-DOPA oxidation.

In each case, a pair of peaks related to the quasi-reversible redox process of L-DOPA may be observed. The anodic peak, well highlighted, corresponds to the L-DOPA oxidation to the quinone derivative with an unmodified lateral chain (Figure 5). The cathodic peak corresponding to the reduction in levodopa has a low intensity, especially in the case of the C-SPCE sensor. A new redox couple becomes more obvious at the subsequent scanning due to the appearance of a slow chemical process, which takes place after the oxidation of L-DOPA. Therefore, an oxidation product is formed, cyclodopa (Figure 6), which is redox-active [43].

The IIa/IIc pair of peaks is due to the oxidation–reduction in the cyclodopa derivative to dopachrome. Thus, the levodopa oxidation mechanism occurs in two stages, each of which involves the transfer of two electrons and two protons [44]. More precisely, CVs show a quasi-reversible two-electron process between levodopa and open-chained dopaquinone. This behavior has been observed in other previous studies reported in the literature [43,45,46,47].

**Figure 7:**
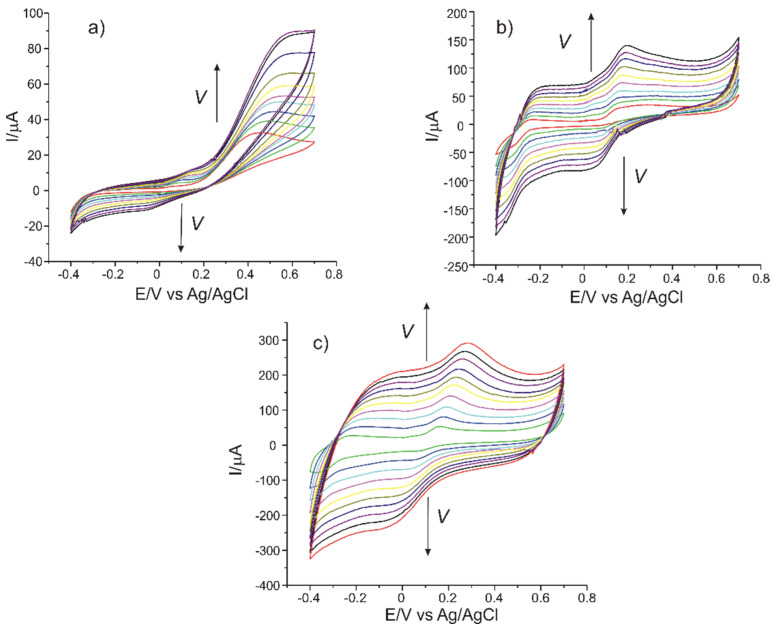
Cyclic voltammograms of (**a**) C-SPCE, (**b**) MC-SPCE and (**c**) OMC-SPCE immersed in 1 × 10^−3^ M L-DOPA solution recorded at various scan rates within the range 0.1–1.0 V·s^−1^.

In the case of C-SPCE, the peaks obtained at the scan rate of 0.1 V·s^−1^ have low intensities and are less visible due to the influence of the capacitive current. At a higher scanning rate, the faradaic currents are higher and the peaks are better defined, as can be seen in Figure 7.

MC-SPCE and OMC-SPCE have higher and better-defined peaks due to the better electrocatalytic capacity of mesoporous carbon, respectively, of organized mesoporous carbon. The volume and organized structure of the pores favor a faster electron transfer, which improves the electrochemical signal. According to the studies in the field, pH plays an essential role in the occurrence of the second redox process, and there is enough unprotected quinone available in the pH used in the present study (7.0) in order to allow the cyclization reaction to take place [43,45].

**Figure 8:**
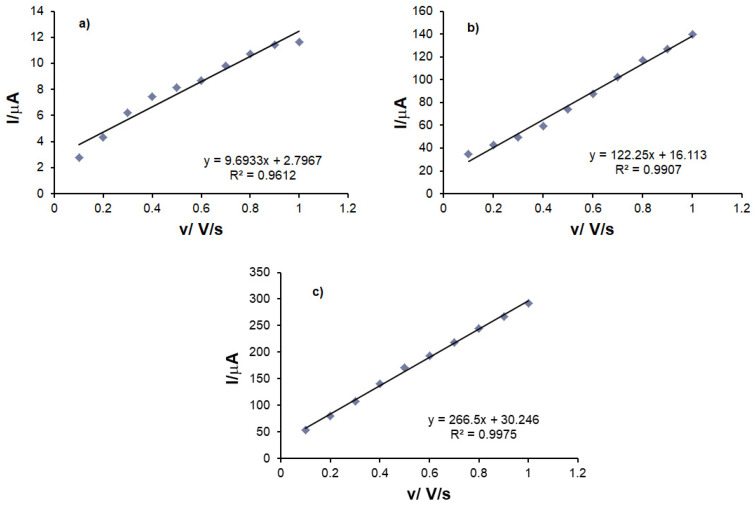
Linear dependence between anodic peak current and scan rate for (**a**) C-SPCE, (**b**) MC-SPCE, (**c**) OMC-SPCE.

The influence of the scan rate on the voltammetric response of the three sensors in the L-DOPA 1.0 × 10^−3^ M-PBS 0.1 M pH = 7.0 solution was studied in the next stage. Scan rates ranged from 0.1 to 1.0 V·s^−1^. The results obtained are shown in Figure 7 and Figure 8.

The currents of the anodic peak I_a_ were plotted as a function of the scan rate. A linear dependence between I_a_ and v was observed in all three cases, which proves that the determining stage of the oxidation–reduction process of L-DOPA is the electron-transfer reaction [48]. The interaction time was 5 s, the same for all measurements. The decrease in current that is observed in cyclic voltammograms after the first redox cycle is due to the adsorption of the oxidation product on the electrode.

The degree of surface coverage with the electroactive species was calculated by using the equation of the dependence between Ia and v and the Laviron equation [18].

(2) $$I_{a}=\frac{n^{2}F^{2}\Gamma Av}{4RT}$$

Table 3 presents the linear dependence equations between I_a_ and v, as well as the values of the degree of coverage of the sensor surface with the electroactive species (Г).

Comparing the results obtained with the three sensors C-SPCE, MC-SPCE and OMC-SPCE, the fact may be observed that the oxidation process controlled by electron transfer is faster in the case of OMC-SPCE, its value being higher for this sensor. 

These results confirm the superior electrochemical properties of organized mesoporous carbon, such as electrical conductivity, chemical inertia, high active surface area and uniform pore size [49].

### 3.4. Preparation of the Calibration Curve

The influence of levodopa concentration on the sensor’s response was studied by cyclic voltammetry. The working electrodes were immersed, one by one, in solutions with different concentrations of analyte and the cyclic voltammograms were recorded. The concentration range studied was 0.1–10.48 µM. Stable and reproducible signals were obtained for all the analyzed solutions by using cyclic voltammetry.

**Figure 9:**
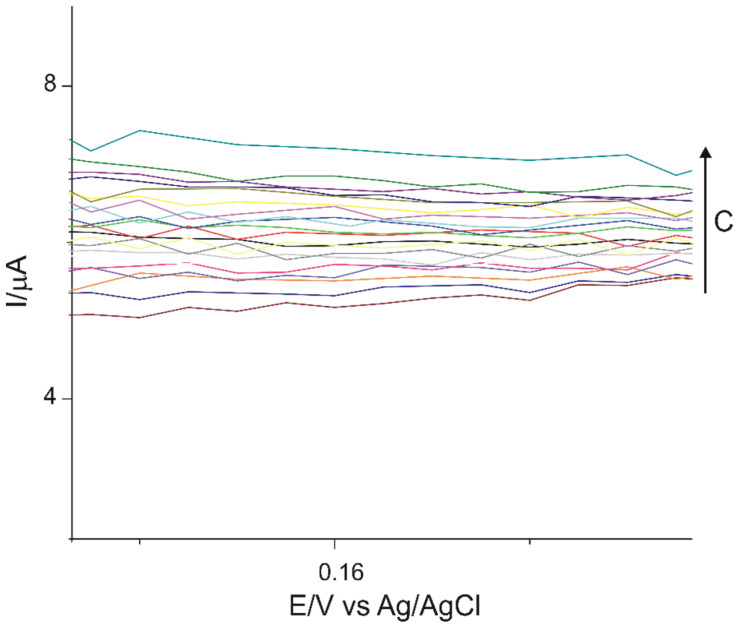
CVs of OMC-SPCE immersed in L-DOPA solution in the concentration range 0.1–1 µM (zoom-in of the anodic peak).

In Figure 9, a zoom-in of the anodic peak obtained with OMC-SPCE immersed in levodopa solutions in the concentration range 0.1–1 µM is presented. The increase of the currents when the concentration increase can be observed. 

**Figure 10:**
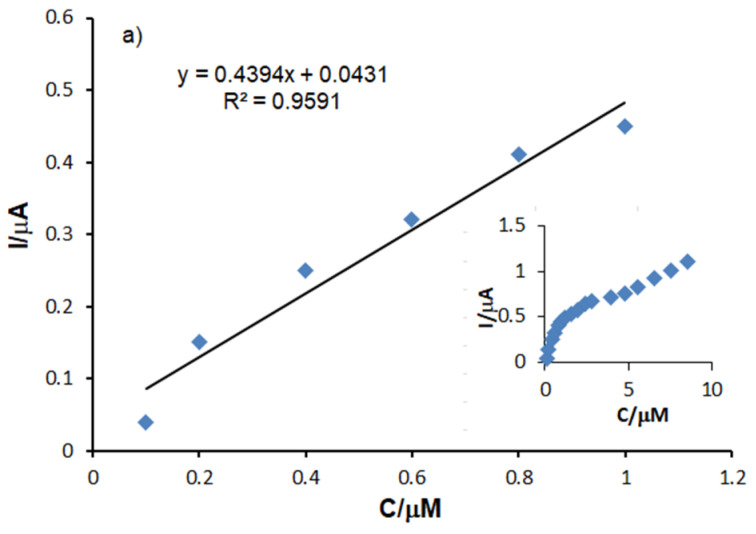

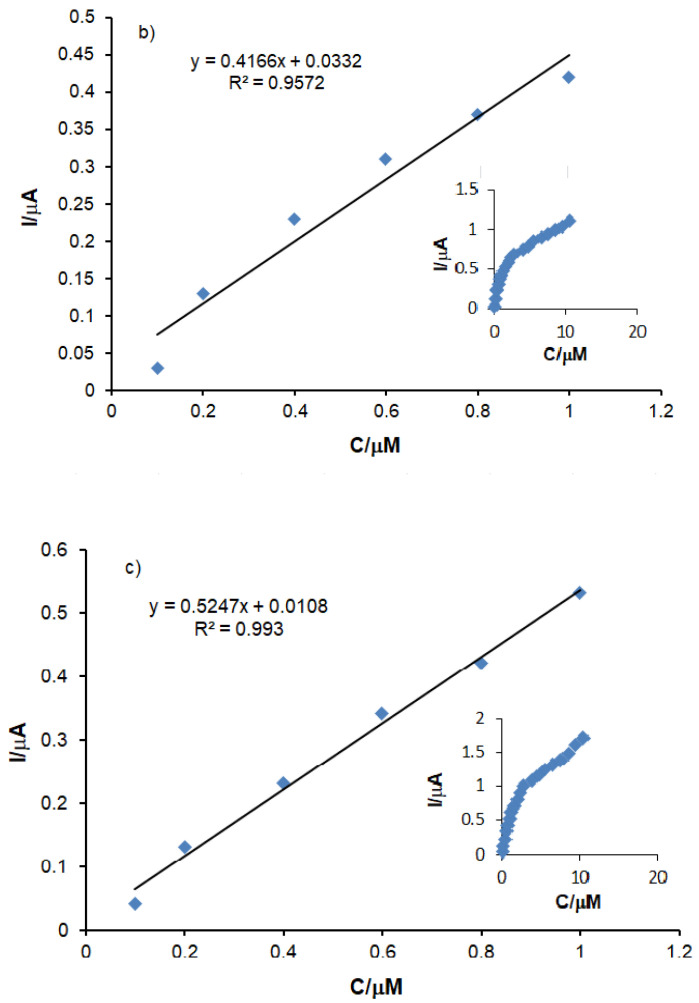
Dependence of the anodic peak as a function of L-DOPA concentration (0.1–1 µM) for (**a**) C-SPCE, (**b**) MC-SPCE, (**c**) OMC-SPCE. The inserted figures show the dependence of the anodic peak depending on the concentration studied throughout the entire concentration range (0.1–10.48 µM).

Figure 10 shows the dependencies between the anodic peak current (the baseline corrected) of the three sensors and the concentration of L-DOPA in the analyzed solutions (support electrolyte PBS of pH 7.0).

A linear increase in I_a_ may be observed throughout the concentration range analyzed, followed by a plateau phase, where the sensor response does not change significantly when the L-DOPA concentration increases. In order to achieve the calibration linear equation, the range of 0.1–1 μM, in which I_a_ increases linearly with the concentration, was selected for all the sensors studied.

The plot illustrating the dependence between the I_a_ values and the concentrations in the 0.1–1 μM range shows a linear dependence and a determination coefficient close to the ideal value 1 for the three sensors. Therefore, the detection and quantification limits were calculated by using the calibration equations corresponding to the concentration range envisaged.

The detection and quantification limits were calculated according to the formula 3 σ/m and 10 σ/m, respectively, where m is the slope of the calibration plot and σ is the standard deviation (*n* = 7) of the voltammetric signals corresponding to the lowest concentration (0.1 µM).

The results obtained for the three sensors are presented in Table 4.

As it may be observed, OMC-SPCE shows the lowest values of the detection and quantification limits, the values increasing in the sequence OMC-SPCE < MC-SCPE < C-SPCE. The higher sensitivity of OMC-SPCE could be related to the larger active surface of the sensor and to the faster electron transfer, favored by the organized structure of the mesoporous carbon. From the presented results, the conclusion could be drawn that the carbon structural change has a major influence on the electroanalytical response, the OMC-SPCE sensor thus having a better sensitivity. The calculated LOD values are generally lower than those obtained by using other modified screen-printed sensors, (see Table 5), which demonstrates the very good sensitivity of these sensors to L-DOPA detection.

The sensors characterized in the present study are sensitive enough to be used in the laboratory for the analysis of L-DOPA from complex samples at different levels of concentration. However, the sensors with better performance characteristics were used in the validation studies at the laboratory level.

### 3.5. Stability, Repeatability Interference Studies

The OMC-SPCE stability was analyzed by cyclic voltammetry, performing a number of 30 measurements and using a 1.0 × 10^−6^ M L-DOPA solution. The results confirmed the fact that the sensor was very stable, with insignificant differences between the currents of the anodic peak.

For the repeatability analysis, the OMC-SPCE sensor was used for 7 consecutive immersions in solutions with the same L-DOPA concentration, the sensor being rinsed before each new recording. The relative standard deviation (RSD) of the anodic peak potential did not exceed 3%.

For the interference analysis, different concentrations of several compounds which may be found in commercial products, along with L-DOPA, were used such as L-tryptophan, L-phenylalanine and L-tyrosine. The results show that the peaks related to the presence of L-DOPA do not undergo significant changes due to the interferences introduced (see Table 6).

The calculated tolerance limit (interference concentration which caused an RSD of approximately 5%) was 2.0 × 10^−5^ M for L-tyrosine and phenylalanine and 5.0 × 10^−5^ M for L-tryptophan. 

The conclusion may be drawn that the OMC-SPCE sensor may be used for the detection of L-DOPA in complex samples containing other amino acids without using separation methods or pretreating the samples to be analyzed.

### 3.6. Levodopa Determination in Real Samples

Taking into consideration its superior characteristics, the OMC-SPCE sensor was used in subsequent analyses in order to quantify L-DOPA in food supplements (Bio Raw Foods, Haya Labs, Now Foods). The products were selected, analyzing their composition and concentration. The solutions obtained after dissolving, homogenizing and filtration of the contents of the capsules were analyzed by cyclic voltammetry. The results obtained by the electrochemical method were compared with those obtained by using the FTIR method. The purpose of the analysis was to demonstrate the feasibility of the voltammetric method and the optimal sensitivity of OMC-SPCE for the quantitative analysis of L-DOPA.

Different amounts were taken from the capsule contents of each food supplement, i.e., 0.015 g, 0.035 g and 0.055 g, which, initially dispersed in the supporting electrolyte (PBS 0.1 M pH = 7.0), homogenized and subsequently filtered. The solutions obtained were analyzed by cyclic voltammetry.

**Figure 11:**
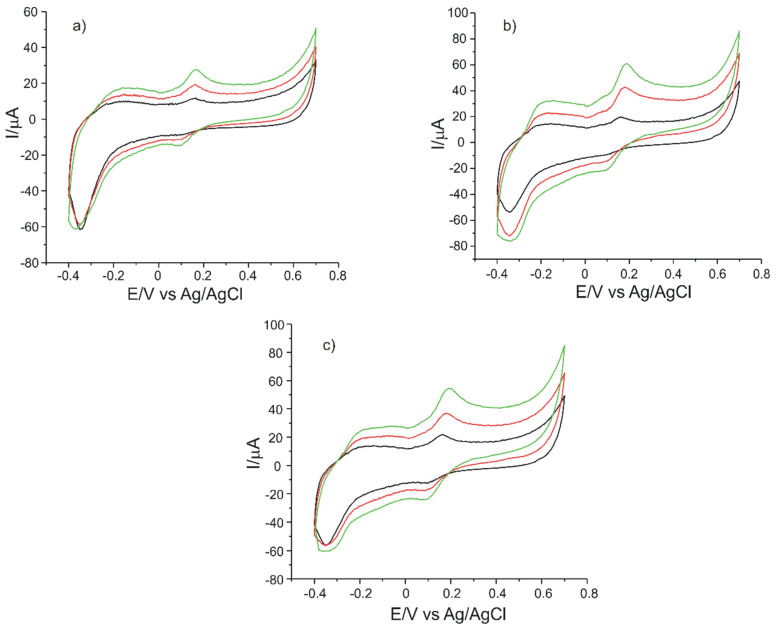
CVs of OMC-SPCE immersed in the products (**a**) Bio Raw Foods, (**b**) Haya Labs, (**c**) Now Foods, of different concentrations: 0.015 g (black line), 0.035 g (red line), 0.055 g (green line). The scan rate was 0.1 V·s^−1^.

Figure 11 shows the CVs of OMC-SPCE immersed in solutions of different concentrations from the Bio Raw Foods, Haya Labs and Now Foods products.

The cyclic voltammograms recorded with OMC-SPCE, for each food supplement, revealed the oxidation and reduction peaks related to the presence of L-DOPA in the solution to be analyzed.

Taking into account the peak current corresponding to the presence of levodopa, the amount of product taken and the equation of the calibration, the concentrations of L-DOPA in the dietary supplements were calculated. This method has been applied in other research studies previously reported [18,37,56,57].

All the analyses were carried out in triplicate. The results obtained are included in Table 7.

The coefficient of variation did not exceed 3.5% for all the measurements, demonstrating the good accuracy of the methods.

**Figure 12:**
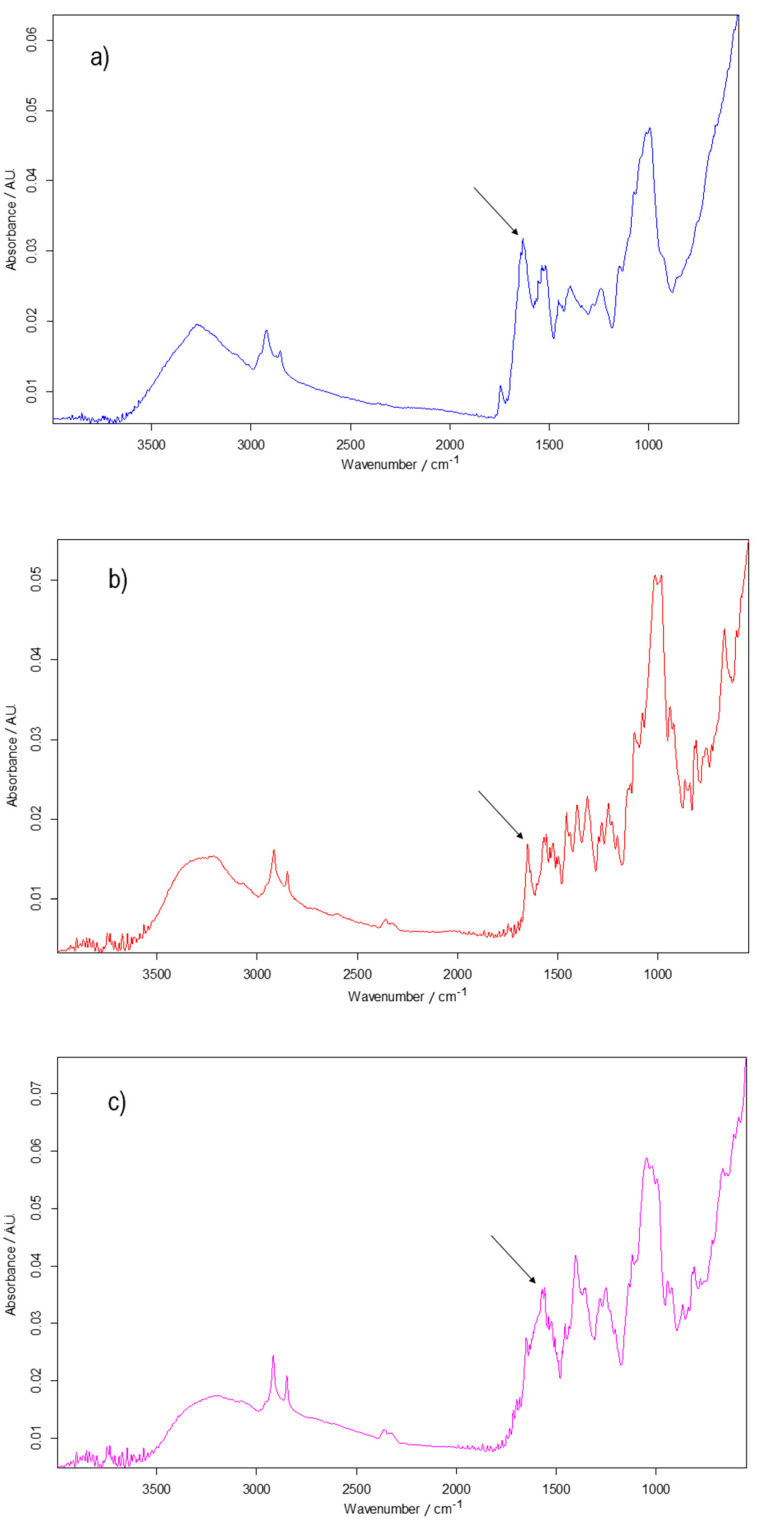
FTIR spectra of the (**a**) Bio Raw foods, (**b**) Haya and (**c**) Now foods samples.

In order to verify the accuracy and feasibility of the voltammetric method, the FTIR spectrometric method was applied. Figure 12 shows the FTIR spectra of the samples from the analyzed food supplements (Bio Raw Foods, Haya Labs and Now Foods).

The samples to be analyzed were mixed with potassium bromide, without any pretreatment stages. A standard sample prepared from pure levodopa and KBr was used, the concentration of L-DOPA being 1%. All experiments were performed in triplicate. The wavenumber related to the vibration of the >C = O group was 1649 cm^−1^ for all the samples [58,59], characteristic of levodopa. Therefore, in the case of this wavenumber, the absorbance was measured for both the standard sample and the samples from the food supplements. These food supplements represented the real samples used for calculating levodopa concentrations. The results obtained are presented in Table 7.

The values obtained by using the FTIR method are similar to those obtained by cyclic voltammetry. Analysis of variance was used in order to quantify the differences among the results obtained. the p-value obtained was 0.97246 and this value is less than the significance level of 0.05, and it can be concluded that the population means are significantly different. This result proves that the electrochemical method based on the screen-printed carbon sensor modified with organized-structure mesoporous carbon is valid for the detection of levodopa in real samples.

## 4. Conclusions

The electrochemical behavior of three modified carbon-based screen-printed sensors (C-SPCE, MC-SPCE, and OMC-SPCE) in different electroactive solutions was studied analyzed in this research work. The obtained electrochemical parameters showed that OMC-SPCE has superior results as compared to the other electrodes used, due to the ordered structure of the mesoporous carbon and superior sensing properties.

The redox behavior of levodopa using the three screen-printed sensors, applying cyclic voltammetry, was studied and the mechanism of detection was clearly demonstrated. 

The calibration curve of the OMC-SPCE towards L-DOPA showed linearity in the concentration range of 0.1–1 μM, and low detection (0.290 μM) and quantification (0.966 μM) limits. Furthermore, OMC-SPCE was successfully used for the quantitative determination of L-DOPA in three food supplements. The voltammetric method quantitative results were validated by using the FTIR spectrometric method, the L-DOPA concentration values obtained being similar and the differences are statistically insignificant. In addition, the OMC-SPCE has shown good stability and repeatability, making this method suitable for the detection of levodopa in food, pharmaceutical or medical samples.

## Figures and Tables

**Table 1:** Electrochemical parameters obtained by the three sensors by immersion in a K_4_[Fe (CN)_6_] 1.0 × 10^−3^ M-PBS 0.1 M solution.

Sensor	I_a_ ^1^ (µA)	I_c_ ^2^ (µA)	I_c_/I_a_	E_a_ ^3^ (V)	E_c_ ^4^ (V)	E_1/2_ ^5^ (V)	ΔE ^6^ (V)
C-SPCE	24.01	−25.99	1.082	0.452	0.003	0.227	0.449
MC-SPCE	42.92	−48.51	1.13	0.275	0.171	0.223	0.104
OMC-SPCE	43.38	−50.00	1.152	0.298	0.155	0.226	0.143

^1^ Anodic peak current; ^2^ cathodic peak current; ^3^ anodic peak potential; ^4^ cathodic peak potential; ^5^ half-wave potential E_1/2_ = (E_a_ + E_c_)/2; ^6^ ΔE = E_a_ − E_c_.

**Table 2:** Linear dependence equation, R^2^, active surface area and roughness factor for the three sensors.

Sensor	I_pa_ vs. v^1/2^	R^2^	A (cm^2^)	Roughness Factor
C-SPCE	I_pa_ (A) = 6.96 × 10^−5^ v^1/2^ (V·s^−1^)^1/2^ + 2.00 × 10^−6^	0.9999	0.0962	0.76603
MC-SPCE	I_pa_ (A)= 1.81 × 10^−4^ v^1/2^ (V·s^−1^)^1/2^ − 1.88 × 10^−5^	0.9962	0.2511	1.99922
OMC-SPCE	I_pa_ (A) = 1.93 × 10^−4^ v^1/2^ (V·s^−1^)^1/2^ − 2.16 × 10^−5^	0.9971	0.2667	2.12365

**Table 3:** The linear equation between I_a_ and v, R^2^, and the concentration of the active species on the surface of the sensors.

Sensor	Linear Equation	R^2^	Г (mol × cm^−2^)
C-SPCE	I = 9.693 × 10^−6^ v + 2.796 × 10^−6^	0.9612	2.45 × 10^−11^
MC-SPCE	I = 1.222 × 10^−4^ v + 1.611 × 10^−5^	0.9907	1.19 × 10^−10^
OMC-SPCE	I = 2.665 × 10^−4^ v + 3.024 × 10^−5^	0.9975	2.43 × 10^−10^

**Table 4:** Calibration linear equations, detection and quantification limits of the three carbon-based sensors.

Sensor	Calibration Linear Equation	LOD (M)	LOQ (M)
C-SPCE	y = 0.4394 x + 0.0431	1.23 × 10^−6^	4.11 × 10^−6^
MC-SPCE	y = 0.4166 x + 0.0332	4.44 × 10^−7^	1.48 × 10^−6^
OMC-SPCE	y = 0.5247 x + 0.0108	2.90 × 10^−7^	9.66 × 10^−7^

y = Ia (μA); x = c (μM).

**Table 5:** Main modified sensors used to determine levodopa with different voltammetric techniques.

Modified Electrode	Detection Technique	Linearity Range/μM	LOD/μM	Ref
FCMPNE ^a^	DPV	2–500	1.200	[50]
Oxovanadium–salen thin film/GPE ^b^	CV	1–100	0.800	[51]
Gold Screen Printed	CV	99–1200	68.000	[52]
Co(DMG)_2_ClPy − MWCNT/BPPG ^c^	CV SWV	3–100	0.860	[53]
PPy − MWCNTs/GCE ^d^	CV	1–100	0.100	[54]
MWCNT/PNB/GCE ^e^	DPV	1–100	0.370	[44]
TNF/GO/GCE ^f^	DPV	0.04–79	0.022	[55]
C-SPCE	CV	0.1–1	1.230	This work
MC-SPCE			0.447	
OMC-SPCE			0.290	

^a^ FCMPNE-Ferrocene Modified Carbon Nanotubes Paste Electrode. ^b^ Graphite-Polyurethane Electrode. ^c^ Basal plane pyrolytic graphite (BPPG) electrode modified with chloro (pyridine) bis (dimethylgyoximato) Cobalt (III). ^d^ PPy-Polypyrrole. ^e^ Glassy carbon electrode modified with Poly (Nile blue-A) (PNB) and multiwalled carbon nanotube. ^f^ Nanofiber/graphite oxide/glassy carbon electrode.

**Table 6:** Interference of chemically-related compounds on the detection of 10^−6^ M concentration levodopa.

Interferent	Interferent Concentration	Recovery/%	RSD/%
L-phenylalanine	1.0 × 10^−5^ M	105.13	3.50
L-tyrosine	1.0 × 10^−5^ M	101.25	1.14
L-tryptophan	1.0 × 10^−5^ M	101.00	0.83

**Table 7:** L-DOPA concentrations obtained from the two analysis methods used in this study.

Dietary Supplement	c% Levodopa FTIR	RSD (%)	c% Levodopa CV	RSD (%)
Bio RawFoods	1.32 ± 0.05	3.45	1.04 ± 0.03	3.23
Haya Labs	2.30 ± 0.07	2.85	2.26 ± 0.06	2.65
Now foods	2.53 ± 0.02	2.78	2.61 ± 0.07	2.75

## Data Availability

The authors confirm that the data supporting the findings of this study are available within the article.
